# Anti-inflammatory, cytotoxicity and antilipidemic properties: novel bioactivities of true cinnamon (*Cinnamomum zeylanicum* Blume) leaf

**DOI:** 10.1186/s12906-022-03728-5

**Published:** 2022-10-04

**Authors:** Walimuni Prabhashini Kaushalya Mendis Abeysekera, Galbada Arachchige Sirimal Premakumara, Wanigasekera Daya Ratnasooriya, Walimuni Kanchana Subhashini Mendis Abeysekera

**Affiliations:** 1 Department of Biosystems Technology, Faculty of Technology, University of Sri Jayewardenepura, Pitipana, Homagama, Sri Lanka; 2 Department of Basic Science and Social Science, Faculty of Nursing, University of Colombo, Colombo, Sri Lanka; 3 Department of Zoology and Environmental Sciences, Faculty of Science, University of Colombo, Colombo, Sri Lanka; 4 Department of Agricultural Technology, Faculty of Technology, University of Colombo, Colombo, Sri Lanka

**Keywords:** Ceylon cinnamon, Leaf extracts, Anti-inflammatory, Growth inhibition & cytotoxicity, Antilipidemic

## Abstract

**Background:**

The leaf of Ceylon cinnamon (true cinnamon) is traditionally claimed for a variety of health benefits. However, reported scientific information is scanty and needs urgent attention for value addition.

**Methods:**

Ethanolic (95%) and Dichloromethane:Methanol (DM, 1:1 v/v) leaf extracts of Ceylon cinnamon were evaluated for a range of medically important bioactivities namely anti-inflammatory [nitric oxide scavenging activity (NOSA), superoxide scavenging activity (SCA), COX1 and COX2 inhibition], growth inhibition & cytotoxicity against MCF7, HePG2 and AN3CA carcinoma cell lines, glutathionase-*S*-transferase (GST) inhibition and antilipidemic (anti-HMG-CoA reductase, anti-lipase, anti-cholesterol esterase, and cholesterol micellization inhibition) properties in vitro (*n* = 3). Further, a range of bioactive compounds in both leaf extracts was also quantified (*n* = 3).

**Results:**

Both leaf extracts had all the investigated bioactive compounds and possessed moderately potent bioactivities compared to the reference drugs used in the study. Ethanolic leaf extract (ELE) exhibited the highest activities (IC_50:_ μg/mL) for NOSA (40.26 ± 0.52), SCA (696.24 ± 40.02), cholesterol esterase inhibition (110.19 ± 1.55), cholesterol micellization inhibition (616.69 ± 7.09), GST inhibition (403.78 ± 2.70) and growth inhibition (GI_50_: 144.84 ± 1.59-269.00 ± 0.51) & cytotoxicity (LC_50_: 355.44 ± 9.38-717.71 ± 23.69) against studied cancer cell lines. In contrast, COX1 & COX2 (IC_50:_ 6.62 ± 0.85 and 44.91 ± 3.06 μg/mL) and HMG-CoA reductase & lipase inhibitory activities (36.72 ± 4.74 and 19.71 ± 0.97% inhibition at 200 and 600 μg/mL) were highest in DM extract. ELE also showed the highest quantities (0.81 ± 0.06-104.38 ± 1.79) of tested compounds (mg/g extract) where eugenol was the highest and gallic acid was the lowest among quantified.

**Conclusion:**

Both leaf extracts of Ceylon cinnamon had all the tested bioactive compounds and possess all the investigated bioactivities. This is the 1st study to report all the investigated bioactivities of the leaf of Ceylon Cinnamon.

## Background

Inflammation is a pervasive form of defense mechanism employed by both innate and adaptive immune systems in the body. It can be either acute or chronic. Acute inflammation is the immediate biochemical and cellular responses to remove the endogenous and exogenous antigens. This immediate response causes to activation of inflammatory mediators and the recruitment of inflammatory cells to the site of inflammation [1, 2]. Chronic inflammation occurs when acute inflammation does not resolve. Although inflammation has a protective role, persistent, dysregulated, and unresolved chronic inflammation is reported to play a crucial role in the onset and development of many chronic diseases such as cancer, atherosclerosis, diabetes, neurodegenerative diseases, and arthritis [3–7].

It is now very well reported that inflammation acts as a gearing force in all stages of carcinogenesis [8]. Several inflammatory mediators, such as cytokines, chemokines, prostaglandins, nitric oxide (NO), leukotrienes, inducible cyclooxygenase (COX2) and inflammatory transcription factors such as NF-κB disrupt the normal signaling cascades within cells and contributes towards the development of neoplasms and cancer progression. Thus, the composition and responses of these inflammatory mediators in the tumor inflammatory microenvironment play a pivotal role in the influence of the disease outcome [6, 8]. Further, it is estimated that nearly 20% of human cancers are related to chronic inflammation [6, 8]. Among a wide range of cancer types breast cancer is the most frequently diagnosed cancer among women worldwide. Globally, liver cancer is the fifth most common malignancy in men and the ninth in women with a poor prognosis and five-year survival rate (< 9%) [9]. Whereas, endometrial cancer (a tumor originating in the endometrium) is the most common gynaecological malignancy in developed countries and the sixth most common cancer in women worldwide [9].

Hyperlipidemia has shown a strong association with cardiovascular diseases in numerous studies conducted in both developed and developing countries and it is the number one cause of death globally [10–13]. Further, it is reported that 18% of global cerebrovascular disease and 56% of global ischaemic heart disease were due to elevated high cholesterol levels. Moreover, nearly 8% of all disease burden in developed countries is due to elevated blood cholesterol [10]. In addition, several studies have shown that hyperlipidemia and inflammation are closely interconnected and have a significant contribution to certain cardiovascular diseases such as atherosclerosis [14, 15]. A recent study has shown that blockade of interleukin (IL)-1b reduces the incidence of cardiovascular events in patients diagnosed with myocardial infarction and C-reactive protein levels > 2 mg/L. The above study strongly confirmed the linkage between lipids and inflammation, since activation of Nod-like receptor protein 3 inflammasome by lipids leads to IL-1b activation [15].

Despite the recent advanced therapies for cancer, hyperlipidemia, and inflammation still, there is no permanent cure for any of these diseases. Therefore, exploration of new drug leads from various natural products is indispensable [16, 17]. In such instances, medicinal herbs and spices with already known medicinal properties in traditional medicine could be a potential source since they are time tested medicines with minimum or no side effects [16, 18, 19]. Cinnamon is a medicinal spice in the genus *Cinnamomum* and there are several *Cinnamomum* species worldwide [20]. However, the current cinnamon trade world over depends particularly on using four *Cinnamomum* species. Within these four species, three *Cinnamomum* species are ‘fake’/Cassia type cinnamons whereas Ceylon cinnamon is the only ‘true cinnamon’ [21]. Sri Lanka is the leading exporter of true cinnamon to the world market contributing 85% market share [21, 22]. The main use of true cinnamon is as a spice and a flavoring agent. However, Ceylon cinnamon has a very long history of being used in the traditional Sri Lankan indigenous system of medicine to treat a variety of diseases [18, 19]. This medicinal property has been scientifically validated mainly using in vitro and in vivo studies both nationally and internationally [20, 23].

The bark is the most investigated part of this medicinal properties and essential oils of both bark and leaf are also studied for some biological activities [20, 23]. However, to date available scientific literature on the medicinal properties of the true cinnamon leaf is extremely scarce even though the leaf is used in number of Sri Lankan medicinal formulations to treat a variety of alignments [18, 19]. We have previously reported antioxidant and anti-diabetic activity of true cinnamon leaf and results indicated high antioxidant activity and moderately potent anti-diabetic activity compared to the bark of Ceylon cinnamon [24, 25]. However, except for our reports on antioxidant and anti-diabetic activities, comprehensive scientific reports regarding the biological activities of true cinnamon leaf is extremely limited. Moreover, there is no previous scientific literature on the anti-inflammatory, growth inhibition and cytotoxicity, GST inhibitory activity and antilipidemic potential of true cinnamon leaf. In this connection, the present study was carried out for a comprehensive investigation of anti-inflammatory, growth inhibition and cytotoxicity, GST inhibitory activity, and antilipidemic potential of true cinnamon leaf to scientifically validate the traditional claims of medicinal properties of the leaf of Ceylon cinnamon.

## Materials and methods

### Materials

#### Sample collection

Fresh cinnamon leaves were collected from disease free healthy cinnamon plants in the cinnamon plantations of L.B spices (Pvt) Ltd., Aluthwala, Galle, Sri Lanka (longitude: 80.1401^0^ E, latitude: 6.1802^0^ N). From the collected leaf samples specimens were prepared (HTS-CIN-1) for future reference and were stored at the Pharmacognosy laboratory of the Herbal Technology Section (HTS) of Industrial Technology Institute (ITI), Sri Lanka. Further, leaf samples (voucher no: CZB-KA) were authenticated by Mr. N.P.T. Gunawardena, Officer-In-Charge, National Herbarium, Department of Botanic Gardens, Peradeniya, Sri Lanka, and were used for the analysis.

#### Preparation of leaf extracts

Collected fresh cinnamon leaves were dried for 7 days at room temperature (30 ± 2 °C) and were powdered. Twenty grams of powdered leaf samples were extracted separately in 200 mL of 95% ethanol in a soxhlet extractor (4-5 h; 4-6 cycles) and 200 mL of dichloromethane:methanol (DM, 1:1 v/v) with periodic shaking for 7 days at room temperature (30 ± 2 °C). Then leaf extracts were separately filtered, evaporated, and freeze dried (Christ-Alpha 1-4 Freeze dryer, Biotech International, Germany). Then extracts were kept under -20 °C until analysis.

#### Chemicals and equipment

Phenazine methosulfate (PMS), nicotinamide adenine dinucleotide (NADH), nitroblue tetrazolium (NBT), sodium nitroprusside, sulphanilamide, N-(1-Naphthyl)ethylenediamine dihydrochloride*,* sulforhodamine-B (SRB), fetal bovine serum (FBS; ATCC-30-2020™), streptomycin/penicillin, dimethyl sulfoxide (DMSO), trypsin/EDTA, paclitaxel, porcine pancreatic lipase (PPL, type II), cholesterol esterase from porcine pancreas, 4-Nitrophenyl butyrate (p-NPB), oleic acid, phosphatidylcholine, cholesterol, sodium taurocholate hydrate, orlistat, epigallocatechingallate (EGCG), epicatechin, cholestyramine resin, eugenol, kaempferol, trans cinnamaldehyde, trans-cinnamic acid, cinnamyl acetate, phorizidin, gallic acid, catechin, 4-hydroxy benzoic acid, rutin and quercetin, were purchased from Sigma-Aldrich, St. Louis, MO, USA. Assay kits namely HMG-CoA reductase (CS 1090) and total cholesterol (BXC0261) were purchased from Sigma-Aldrich, USA and Fortress diagnostics, UK. Powdered Dulbecco’s Modified Eagle Medium (DMEM) was purchased from Invitrogen Life Technologies, Carlsbad, CA, USA. Human oestrogen positive breast cancer cells (MCF 7; ATCC-HTB-22TM) were purchased from American type cell culture Manassas, USA and human endometrial carcinoma cells (AN3CA; ATCC-HTB-111) were purchased from the American type culture collection in Rockville, MD, USA. Human hepatocarcinoma cells (HepG2; catalogue no. 85011430) were purchased from ECACC, Salisbury, UK. All other chemicals and reagents used for the analysis were of analytical grade or cell culture tested. All the analysis was carried out using 96-well microplate readers (SPECTRAmaxPLUS^384^, Molecular Devices, USA; ELx 800 Universal Micro Plate Reader, BIO-TEK instruments, USA) and morphological changes of the each cancer cells and cells treated with different concentrations of leaf extracts were observed using a phase contrast microscope (Olympus CKX41SF, Japan).

## Methods

### Anti-inflammatory assays

#### Nitric oxide radical scavenging assay

The nitric oxide radical scavenging activity was performed according to the method of Sreejayan and Rao [26] with minor modifications using a microplate reader. Briefly, a reaction volume (100 μL) containing different concentrations of leaf extracts (15.62, 31.25, 62.5, 125, 250 μg/mL) of Ceylon cinnamon and 50 μL of 10 mM sodium nitroprusside in 0.1 M phosphate buffer was incubated at 25 °C in 96 well microplates for 2 hours. After the incubation period, 60 μL of 1% sulfanilamide and 1% N*-*(1-Naphthyl)ethylenediamine dihydrochloride were added to each well, mixed, and incubated again at 25 °C for 30 min. Then absorbance readings were taken at 550 nm. Rutin was used as the positive control.

#### Superoxide radical scavenging assay

Superoxide radical scavenging activity was according to the method of Liu and Ng [27] with slight modifications. Superoxide anions were generated through the non-enzymatic reaction of phenazine methosulfate (PMS), nicotinamide adenine dinucleotide (NADH), and oxygen. Then generated superoxide anions were assayed by the reduction of nitroblue tetrazolium (NBT). Assay volume of 200 μL consisting of 0.2 mM NADH, 0.08 mM NBT and different concentrations of leaf extracts of Ceylon cinnamon in 100 mM phosphate buffer (37.5, 75, 150, 300, 600 μg/mL) were pre read at 560 nm. The reaction was initiated by adding 0.008 mM PMS and was incubated for 10 min at room temperature. After 10 mins absorbance readings were monitored at 560 nm. The positive control, quercetin was studied as the reference drug.

#### COX1 and COX2 enzyme inhibitory assays

COX1 (ovine) and COX2 (human recombinant) enzyme inhibition were determined by enzymes immunoassay (EIA) kit (Catalogue No.560101, Cayman Chemical, Ann Arbor, MI, USA) as per the manufacturer’s instructions. Assay volume of 1 mL containing 960 μL of reaction buffer (0.1 M Tris-HCl, pH -8 containing 5 mM EDTA and 2 mM phenol), 10 μL of COX-1/COX-2 enzyme, 10 μL of heme and 10 μL of extracts of leaf in DMSO (COX1: 25, 50, 100, 200 μg/mL; COX2: 50, 100, 200, 400 μg/mL) were incubated at 37 °C for 10 min. After the incubation period, 10 μL of arachidonic acid (substrate) was added and incubated again at 37 °C exactly for 5 min. Then reaction was stopped by adding 50 μL of 1 M HCl and then 100 μL of stannous chloride was added. The PGF_2α_, produced from the PGH_2_ by reduction with stannous chloride was measured by enzyme immunoassay. Percent inhibition of COX1 and COX2 enzyme activities by leaf extracts of Ceylon cinnamon were calculated compared to control incubations.

### Growth inhibition and cytotoxicity via cell line-based assays

#### Cell culture maintenance

Human oestrogen positive breast cancer cells (MCF 7), human hepatocarcinoma cells (HepG2) and human endometrial carcinoma cells (AN3CA) were grown separately in monolayer cultures in Dulbecco’s Modified Eagle Medium (DMEM) supplemented with 10% fetal bovine serum, 50 IU/mL penicillin and 50 μg/mL streptomycin at 37 °C in a humidified atmosphere (95%) containing 5% CO_2._ Cells were subcultured every 2–3 days, upon reaching 80% confluence.

#### Growth inhibition and cytotoxicity via SRB assay

Growth inhibition and cytotoxicity against selected human carcinoma cell lines namely breast carcinoma (MCF 7), hepatocarcinoma (HepG2) and endometrial carcinoma (AN3CA) cells were evaluated using sulforhodamine-B cytotoxicity assay [28–30]. Each cancer cells (MCF 7, HepG2 and AN3CA) were separately plated in 96-well plates (5000 cells/well) and incubated at 37 °C for 24 h in a humidified environment (95% air and 5% CO_2_). Then varying concentrations such as 25, 50, 100, 200 and 400 μg/mL of both extracts of leaf were separately added and incubated again at 37 °C for 48 h in the same environment (95% air and 5% CO_2_). Paclitaxel was used as the positive control (0.62, 1.25, 2.5, 5 and 10 μg/mL). At the end of the incubation periods cells were fixed with 50 μL of ice-cold 50% trichloroacetic acid and incubated at 4 °C for 60 mins. Then plates were washed five times with distilled water, allowed to dry overnight and 100 μL of SRB solution (0.4% wt/vol in 1% acetic acid) was added to each well. After 30 min, the unbound SRB was removed by washing with 1% acetic acid, and air-dried at room temperature (28 ± 2 °C). The protein-bound stain was solubilized with 200 μL of 10 mM Tris base (pH = 10.2) and plates were shaken for 1 h at room temperature using a plate shaker. Absorbance was measured at 515 nm using a microplate reader. The absorbance of the appropriate blanks and controls (without sample), were used to calculate the growth inhibition/cytotoxicity of the test samples. The growth inhibition/cytotoxicity of extracts and the standard paclitaxel are represented as GI_50_ (concentration of the extracts/paclitaxel at 50% growth inhibition compared to control), LC_50_ (lethal concentration of extract required to kill 50% of cancer cells) and TGI: Total Growth Inhibition (the concentration of extract required to completely halt the growth of cancer cells) values.

### Glutathione S-transferase enzyme inhibition assay

Glutathione *S*-transferase enzyme inhibition assay was performed according to the method of Habig et al. [31]. Briefly, 1 mL of reaction mixture containing 1 mM CDNB, 5 mM GSH and varying assay concentrations of extracts of leaf such as 31.25, 62.5, 125, 250, 500 μg/mL (*n* = 6 each) in 100 mM phosphate buffer (pH 6.5) were mixed well. Then reaction was initiated by the addition of 50 mU of GST enzyme in phosphate buffer. Glutathione *S*-transferase enzyme activity was monitored spectrophotometrically at 340 nm for the formation of thioether by the reaction of glutathione (GSH) with 1-chloro-2, 4-dinitrobenzene (CDNB) for a period of 10 min at 25 °C and inhibition of enzyme was calculated with compared to the control.

### Antilipidemic assays

#### HMG-CoA reductase inhibition assay

Inhibitory activity of leaf extracts on HMG-CoA reductase enzyme was based on the method in our recent publication, Abeysekera et al. [21] via HMG-CoA reductase assay kit (Sigma CS 1090). Varying assay concentrations of ethanolic and DM leaf extracts (100, 150 and 200 μg/mL, *n* = 3 each) were studied for HMG-CoA reductase inhibition. As positive control a clinical drug, pravastatin was studied (0.02 - 1.25 μg/mL, *n* = 3).

#### Lipase inhibition assay

Lipase enzyme inhibition assay was performed by the method of our recent publication Abeysekera et al. [21], a slightly modified method of Kim et al. [29]. A range of varying assay concentrations of ethanolic and DM extracts of leaf (37.5, 75, 150, 300, 600 μg/mL, *n* = 3 each) were evaluated for anti-lipase activity. As the positive control a clinical drug, orlistat was studied (0.20 – 6.25 μg/mL, *n* = 3).

#### Cholesterol esterase inhibition assay

Pancreatic cholesterol esterase inhibition assay was too carried out by the method of our recent publication Abeysekera et al. [21], a minor modified method of Pietsch and Gütschow [30]. Varying assay concentrations such 25, 50, 100, 200 and 400 μg/mL of ethanolic and DM extracts of leaf (*n* = 4 each) were studied. As the positive control a clinical drug, simvastatin was studied (2.5 **-** 30 μg/mL, *n* = 3).

#### Cholesterol micellization inhibition assay

Inhibition of cholesterol micellization by leaf extracts were too based on the method in our recent publication Abeysekera et al. [21], a slightly modified method of Kirana et al. [31]. Both leaf extracts were investigated for cholesterol micellization inhibition using 250, 500 and 1000 μg/mL assay concentrations (*n* = 6 each). As the positive control EGCG was studied (250, 500 and 1000 μg/mL, *n* = 3).

### Quantification of selected compounds

Ten selected compounds including seven phenolic (catechin, eugenol, kaempferol, phorizidin, epicatechin, 4-hydroxy benzoic acid and gallic acid; *n* = 3 each) and three non phenolic compounds (cinnamyl acetate, cinnamaldehyde and trans cinnamic acid; *n* = 3 each) were quantified by HPLC method (SHIMADZU, Kyoto, Japan). The HPLC system was coupled with LC-10ADVP pump and SPD-M10AVP diode array detector. The reverse phase chromatographic column (C 18 Kinetex® 5 μm, Phenyl-Hexyl 100 Å pore size, length 250 mm, internal diameter 4.6 mm; Phenomenex,Torrance, CA, USA) was used in the quantification of compounds. The mobile phase was prepared using 2% (v/v) acetic acid in water (eluent A) and methanol in acetonitrile (4.5/4.0, v/v; eluent B). The binary gradient system was used in the quantification of compounds and which was 20-37% B (5 min), 37-55% B (7 min), 55-63% B (8 min), 63-37% B (1 min) and 37% B (8 min). For a single analysis 35 mins run time was used. The inject volume of all reference standards and samples was 20 μL. The flow rate of the instrument was 1.0 mL/min. The absorption spectrum was scanned within the range of 200–600 nm. The chromatography peaks of ethanolic and DM leaf extracts were confirmed by comparing their retention time with those of reference standards.

#### Statistical analysis

Data of each experiment were statistically analyzed using SAS version 6.12. One way analysis of variance (ANOVA) and the Duncan’s Multiple Range Test (DMRT) were used to determine the differences among treatment means. IC_50_ values were calculated by linear regression analysis computed using excel software. The Pearson’s correlation coefficient was used for the correlation analysis. *P* < 0.05 was regarded as significant.

## Results

### Anti-inflammatory activity

#### Nitric oxide radical scavenging activity

**Fig. 1:**
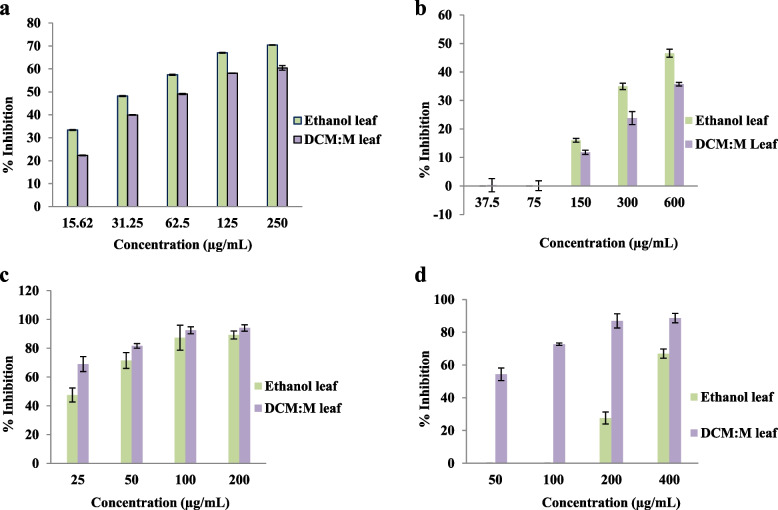
**a** Nitric oxide radical scavenging activity. Results expressed as mean ± SE (*n* = 4 each). IC_50_ ethanolic leaf and DM leaf: 40.26 ± 0.52^b^ & 69.63 ± 0.56^a^ μg/mL respectively. Mean IC_50_ values superscripted by different letters are significantly different at *p* < 0.05. Ethanolic leaf and DM leaf r^2^ = 0.99 and 0.97 respectively. IC_50_ rutin: 17.62 ± 0.01 μg/mL. DM: dichloromethane: methanol. **b** Superoxide radical scavenging activity. Results expressed as mean ± SE (*n* = 4 each). IC_50_ ethanolic leaf and DM leaf: 696.24 ± 40.02^b^ & 1381.42 ± 98.30^a^ μg/mL respectively. Mean IC_50_ values superscripted by different letters are significantly different at *p* < 0.05. Ethanolic leaf and DM leaf r^2^ = 0.95 and 0.94 respectively. IC_50_ quercetin: 75.58 ± 1.97 μg/mL. DM: dichloromethane: methanol; at 37.5 & 75 μg/mL: ethanol leaf no inhibition. **c** COX1enzyme inhibitory activity. Results expressed as mean ± SE (*n* = 3 each). IC_50_ ethanolic leaf and DM leaf: 26.58 ± 2.79^a^ & 6.62 ± 0.85^b^ μg/mL respectively. Mean IC_50_ values superscripted by different letters are significantly different at *p* < 0.05. Ethanolic leaf and DM leaf r^2^ = 0.99. DM: dichloromethane: methanol. **d** COX2 enzyme inhibitory activity. Results expressed as mean ± SE (*n* = 3 each). IC_50_ ethanol leaf and DM leaf: 318.74 ± 12.34^a^ & 44.91 ± 3.06^b^ μg/mL respectively. Mean IC_50_ values superscripted by different letters are significantly different at *p* < 0.05. Ethanol leaf and DM leaf r^2^ = 0.99. DM: dichloromethane: methanol

Investigated leaf extracts including both ethanolic and DM exhibited scavenging of nitric oxide radicals in a dose dependent manner (ethanolic leaf and DM leaf r^2^: 0.99 and 0.97 respectively). Further, compared to the reference drug rutin activities were moderate (IC_50_ 17.62 ± 0.01 μg/mL). Moreover, ethanolic leaf extract showed significantly (*p* < 0.05) high activity (IC_50_ 40.26 ± 0.52 μg/mL) than DM leaf extract (IC_50_ 69.63 ± 0.56 μg/mL). The dose response relationship of leaf extracts for nitric oxide radical scavenging is given in Fig. 1a.

#### Superoxide radical scavenging activity

Superoxide radical scavenging activity of ethanolic and DM leaf extracts were dose dependent (ethanol leaf and DCM:M leaf r^2^: 0.95 and 0.94 respectively) and moderate compared to the standard drug quercetin (IC_50_ 75.58 ± 1.97 μg/mL). However, ethanolic leaf extract demonstrated significantly (*p* < 0.05) high activity (IC_50_ 696.24 ± 40.02 μg/mL) than DM leaf extract (IC_50_ 1381.42 ± 98.30 μg/mL). The dose response relationship of leaf extracts for superoxide radical scavenging activity is given in Fig. 1b.

#### COX1 and COX2 enzyme inhibitory activity

Both ethanolic and DM leaf extracts exhibited both COX1 and COX2 enzyme inhibition and were dose dependent (COX1: ethanolic leaf and DM leaf r^2^ = 0.99; COX2: ethanolic leaf and DM leaf r^2^ = 0.99). However, both leaf extracts possessed significantly high (*P* < 0.05) COX1 enzyme inhibitory activity than COX2 enzyme inhibitory activity. The IC_50_ values of ethanolic and DM leaf extracts for COX1 and COX2 enzyme inhibitory activities were 26.58 ± 2.79 and 6.62 ± 0.85 μg/mL and 318.74 ± 12.34 and 44.91 ± 3.06 μg/mL respectively. Further, the dose response relationship of leaf extracts for COX1 and COX2 enzyme inhibition are given in Fig. 1c and d respectively.

### Growth inhibition and cytotoxicity via cell line-based assays

Investigated leaf extracts possessed growth inhibition (GI) and cytotoxicity (CT) for human breast carcinoma (MCF 7), hepatocarcinoma (HepG2) and endometrial carcinoma (AN3CA) cells. The demonstrated GI and CT of both leaf extracts towards all carcinoma cells (MCF 7, HepG2 and AN3CA) were dose dependent (ethanol leaf and DCM:M leaf: r^2^ for MCF 7, HePG2 and AN3CA cell lines: 0.97 & 0.98, 0.96 & 0.96 and 0.97 & 0.96 respectively). Further, ethanolic extract of leaf possess significantly high (*p* < 0.05) growth inhibition and cytotoxicity (except GI and CT of AN3CA cells) compared to DM leaf extract. The order of potency of ethanolic leaf extract against GI and CT of carcinoma cell lines were HePG2 > MCF 7 > AN3CA. In contrast, the order of potency of DM leaf extract against GI and CT of carcinoma cell lines were HePG2 > MCF 7 > AN3CA. Nonetheless, both leaf extracts showed highest GI and CT towards HePG2 carcinoma cells. Moreover, both leaf extracts showed moderate GI and CT for all carcinoma cell lines compared to the reference drug paclitaxel. The results of GI, CT and total growth inhibition of ethanolic and DM leaf extracts and paclitaxel on MCF 7, HepG2 and AN3CA carcinoma cells are given in (Tables 1 and 2) respectively.

**Table 1:** Growth inhibition and cytotoxicity on selected human carcinoma cell lines

Carcinoma cell line	Extract	% Net growth					GI _**50**_	LC _**50**_	TGI
		Concentration (μg/mL)							
		25	50	100	200	400			
**MCF 7**	**Ethanolic leaf**	72.75 ± 0.56	73.73 ± 0.59	66.38 ± 0.82	32.09 ± 1.03	2.99 ± 0.47	**152.93 ± 0.99**^**b**^	**650.99 ± 3.43**^**b**^	**401.96 ± 1.83**^**b**^
	**DM leaf**	97.92 ± 0.85	97.49 ± 0.15	89.01 ± 1.14	73.36 ± 0.27	25.75 ± 1.34	**291.20 ± 4.41**^**a**^	**801.21 ± 14.50**^**a**^	**546.22 ± 9.36**^**a**^
**HePG2**	**Ethanolic leaf**	99.75 ± 1.96	97.16 ± 1.69	68.46 ± 2.86	24.67 ± 2.22	−17.12 ± 9.04	**144.84 ± 1.59**^**b**^	**355.44 ± 9.38**^**b**^	**259.91 ± 12.60**^**b**^
	**DM leaf**	99.55 ± 1.76	96.00 ± 5.27	94.32 ± 4.35	36.60 ± 3.54	−11.27 ± 5.78	**197.16 ± 8.85**^**a**^	**516.85 ± 26.65**^**a**^	**357.01 ± 17.24**^**a**^
**AN3CA**	**Ethanolic leaf**	99.19 ± 0.11	96.59 ± 1.12	91.05 ± 4.03	73.73 ± 2.80	16.11 ± 2.98	**269.00 ± 9.51**^**a**^	**717.71 ± 23.69**^**a**^	**493.36 ± 16.41**^**a**^
	**DM leaf**	97.89 ± 1.11	92.79 ± 2.94	86.97 ± 2.96	72.91 ± 3.80	10.69 ± 2.73	**251.70 ± 9.76**^**a**^	**680.12 ± 15.70**^**a**^	**470.93 ± 16.94**^**a**^

Results expressed as mean ± SE. (*n* = 4 each). Ethanolic leaf and DM leaf: r^2^ for MCF 7, HePG2 and AN3CA cell lines: 0.97 & 0.98, 0.96 & 0.96 and 0.97 & 0.96 respectively. GI_50_, LC_50_ and TGI values superscripted by different letters within ethanolic and DM leaf extracts for each cell line are significantly different at *p* < 0.05; LC_50_ values of ethanolic and DM leaf extracts for each cell line were calculated by extrapolating the graph

*GI*_*50 *_concentration of extract at 50% inhibition of cancer cells growth compared to control, *LC*_*50 *_concentration of extract which kills 50% of cancer cells, *TGI* the concentration of extract which halt the cancer cells growth completely, *DM *Dichloromethane Methanol, *TGI* Total Growth Inhibition

**Table 2:** Growth inhibition and cytotoxicity of paclitaxel on selected human carcinoma cell lines

Carcinoma cell line	% Net growth					GI _**50**_	LC _**50**_	TGI
	Concentration (μg/mL)							
	0.62	1.25	2.5	5	10			
**MCF 7**	62.91 ± 5.15	35.20 ± 3.09	7.28 ± 1.13	−95.49 ± 1.82	−97.98 ± 0.87	**1.01 ± 0.04**	**3.82 ± 0.03**	**2.41 ± 0.02**
**HePG2**	69.54 ± 1.94	50.06 ± 0.95	23.44 ± 6.21	−92.97 ± 2.39	−92.97 ± 1.27	**1.34 ± 0.04**	**4.03 ± 0.08**	**2.68 ± 0.06**
**AN3CA**	7.22 ± 1.49	−4.10 ± 3.94	−33.98 ± 1.51	−37.05 ± 0.98	**–**	**0.16 ± 0.01**	**4.91 ± 0.55**	**0.96 ± 0.08**

Results expressed as mean ± SE. (*n* = 3 each); MCF 7, HePG2 and AN3CA: r^2^ = 0.99, 0.98 and 0.92 respectively; − 10 μg/mL concentration didn’t study for the AN3CA cell line

*GI*_*50*_ concentration of extract at 50% inhibition of cancer cells growth compared to control, *LC*_*50*_ concentration of extract which kills 50% of cancer cells, *TGI* Total Growth Inhibition: the concentration of extract which halt the cancer cells growth completely

### Glutathione S-transferase enzyme inhibitory activity

Studied both leaf extracts possessed significant glutathione *S-*transferase enzyme inhibitory activity (*p* < 0.05). However, demonstrated activities were moderate in comparison to the caffeic acid, the standard drug investigated in the study (IC_50_ 205.23 ± 2.27 μg/mL). Further, ethanolic leaf extract evident for the highest activity (IC_50 _403.78 ± 6.04 μg/mL) than the DM leaf extract (only 27.31 ± 2.61% inhibition at 500 μg/mL). The dose response relationship for enzyme inhibition is given in (Table 3).

**Table 3:** Glutathione *S*–transferase enzyme inhibition

Extract	% Inhibition					IC _**50**_ μg/mL
	Concentration (μg/mL)					
	31.25	62.5	125	250	500	
Ethanolic leaf	7.48 ± 0.65	16.78 ± 2.00	31.08 ± 1.82	45.24 ± 0.67	53.58 ± 0.20	**403.78 ± 2.70**
DM leaf	3.47 ± 0.20	6.74 ± 0.42	11.11 ± 1.26	13.28 ± 1.21	27.31 ± 2.61	**–**

Results expressed as mean ± SE (*n* = 6); r^2^: ethanolic leaf and DM leaf = 0.84 & 0.97 respectively; IC_50_ Caffeic acid: 205.23 ± 2.27 μg/mL; DM: dichloromethane:methanol

### Antilipidemic activity

#### HMG-CoA reductase enzyme inhibition

**Fig. 2:**
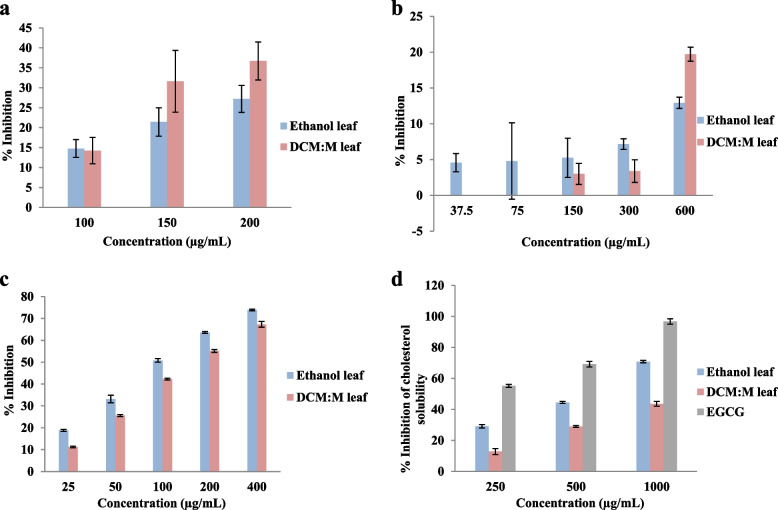
**a** HMG-CoA reductase inhibitory activity. Results expressed as mean ± SE (*n* = 3 each). Ethanolic leaf and DM leaf r^2^ = 1.00. IC_50_ pravastatin: 0.50 ± 0.05 μg/mL. DM: dichloromethane: methanol. **b** Anti-lipase activity. Results expressed as mean ± SE (*n* = 3 each). Ethanolic leaf and DM leaf r^2^ = 0.97 and 0.93 respectively. IC_50_ orlistst: 26.78 ± 2.45 μg/mL. DM: dichloromethane: methanol; at 37.5 & 75 μg/mL: DM leaf no inhibition. **c** Anti-cholesterol esterase activity. Results expressed as mean ± SE (*n* = 4). IC_50_ ethanolic leaf and DM leaf: 110.19 ± 1.55^a^ & 160.74 ± 3.93^b^ μg/mL respectively. Mean IC_50_ values superscripted by different letters are significantly different at *p* < 0.05. Ethanolic leaf and DM leaf r^2^ = 0.99 and 1.00 respectively. IC_50_ simvastatin: 18.56 ± 0.68 μg/mL. DM: dichloromethane: methanol. **d** Cholesterol micellization inhibitory activity. Results expressed as mean ± SE (ethanolic leaf and DM leaf *n* = 6 each; EGCG *n* = 3). IC_50_ ethanolic leaf, DM leaf & EGCG: 616.69 ± 7.09^b^, 1141.66 ± 48.30^a^ & 150.98 ± 18.72^c^ μg/mL respectively. IC_50_ values in a column superscripted by different letters are significant different at *p* < 0.05. EGCG, ethanolic leaf and DM leaf r^2^ = 1.00, 1.00 and 0.95 respectively. DM: dichloromethane: methanol; EGCG: Epigallocatechin gallate

Ethanolic and DM leaf extracts demonstrated HMG-CoA reductase inhibitory activity in a dose dependent manner (ethanolic leaf and DM leaf r^2^ = 1.00). The mean % inhibition of HMG-CoA reductase enzyme by ethanolic and DM leaf extracts were in the range of 14.76 ± 2.23 - 27.23 ± 3.39 and 14.24 ± 3.32 - 36.72 ± 4.74 respectively. Further, both extracts showed moderate HMG-CoA reductase inhibition compared to the standard drug pravastatin (IC_50_ 0.50 ± 0.05 μg/mL). The dose response relationship of ethanolic and DM leaf extracts for HMG-CoA reductase inhibitory activity are given in Fig. 2a.

#### Anti-lipase activity

Both extracts of leaf possessed lipase enzyme inhibition and it was dose dependent (ethanolic leaf and DM leaf r^2^ = 0.97 and 0.93 respectively). The mean % inhibition of lipase enzyme by ethanolic and DM leaf extracts were in the range of 4.56 ± 1.28 - 12.92 ± 0.78 and − 1.40 ± 0.33 - 19.71 ± 0.97 respectively. Compared to the clinical drug orlistat (IC_50_ 26.78 ± 2.45 μg/mL) both leaf extracts had moderate lipase enzyme inhibition. The dose response relationship of ethanolic and DM leaf extracts for lipase enzyme inhibition is given in Fig. 2b.

#### Anti-cholesterol esterase activity

Significant (*p* < 0.05) and dose dependent (ethanolic leaf and DM leaf r^2^ = 0.99 and 1.00 respectively) anti-cholesterol esterase activity was observed in investigated leaf extracts. However, ethanolic leaf extract showed significantly (*p* < 0.05) high activity (IC_50_ 110.19 ± 1.55 μg/mL) compared to DM leaf extract (IC_50_ 160.74 ± 3.93 μg/mL). Compared to the clinical drug simvastatin (IC_50_ 18.56 ± 0.68 μg/mL) both leaf extracts showed moderate cholesterol esterase enzyme inhibition. The dose response relationship of leaf extracts for cholesterol esterase enzyme inhibition is given in Fig. 2c.

#### Cholesterol micellization inhibitory activity

Cholesterol micellization inhibition of leaf extracts and the standard drug EGCG are given in Fig. 1d. Both leaf extracts and EGCG showed dose dependent cholesterol micellization inhibition (ethanolic leaf, DM leaf and EGCG r^2^ = 1.00, 0.95 and 1.00 respectively). However, compared to the standard drug EGCG (IC_50_ 150 ± 10 μg/mL) both leaf extracts had moderate cholesterol micellization inhibitory activity. Further, ethanolic leaf extract demonstrated significantly (*p* < 0.05) high activity (IC_50_ 616.69 ± 7.09 μg/mL) compared to DM leaf extract (IC_50_ 1141.66 ± 48.30 μg/mL). The order of potency of leaf extracts and EGCG for cholesterol micellization inhibitory activity were EGCG > ethanol leaf > DM leaf. The dose response relationship leaf extracts and EGCG for cholesterol micellization inhibition is given in Fig. 2d.

### Quantification of selected compounds

**Fig. 3:**
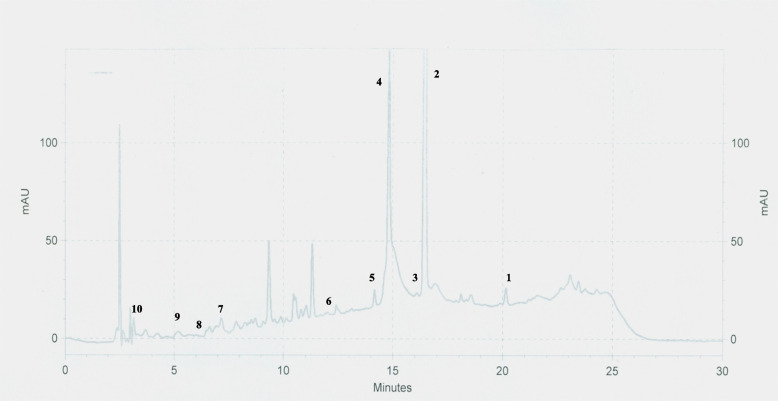
Chromatogram of ethanolic leaf extract. 1: Cinnamyl acetate; 2: Eugenol; 3: Kaempferol; 4: Cinnamaldehyde; 5: Trans cinnamic acid; 6: Phlorizidin; 7: Epicatechin; 8: 4-Hydroxybenzoic acid; 9: Catechin; 10: Gallic acid

**Fig. 4:**
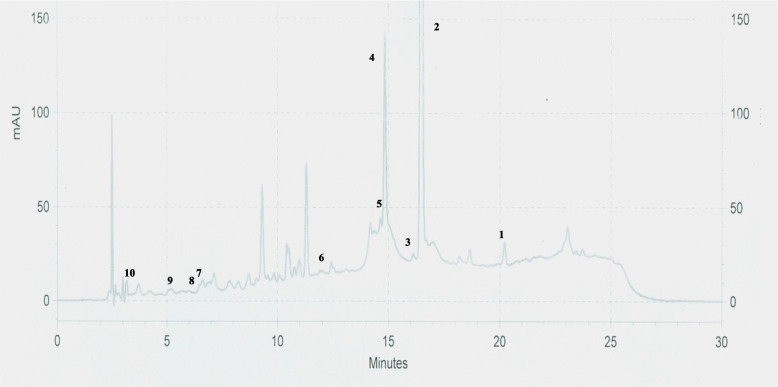
Chromatogram of DM leaf extract. 1: Cinnamyl acetate; 2: Eugenol; 3: Kaempferol; 4: Cinnamaldehyde; 5: Trans cinnamic acid; 6: Phlorizidin; 7: Epicatechin; 8: 4-Hydroxybenzoic acid; 9: Catechin; 10: Gallic acid

Varying quantities of individual phenolic and non phenolic compounds were observed in ethanolic and DM leaf extracts. The quantity of individual compounds in ethanolic and DM leaf extracts ranged from 0.81 ± 0.06 - 104.38 ± 1.79 and 0.76 ± 0.04 - 93.11 ± 0.42 mg/g of extract respectively. Among tested individual compounds ethanolic leaf extract had significantly (*p* < 0.05) high quantities of cinnamyl acetate (44.53 ± 3.22 mg/g of extract), eugenol (104.38 ± 1.79 mg/g of extract), cinnamaldehyde (8.20 ± 0.25 mg/g of extract), trans cinnamic acid (7.68 ± 0.55 mg/g of extract) and phlorizidin (3.85 ± 0.05 mg/g of extract). Whereas DM leaf had significantly (*p* < 0.05) high quantity of epicatechin (10.08 ± 0.07 mg/g of extract). On the other hand kaempferol, catechin, 4-hydroxy benzoic acid and gallic acid contents in ethanolic and DM leaf extracts were statistically in significant (*p* > 0.05). Among tested non phenolics, cinnamyl acetate (ethanolic leaf: 44.53 ± 3.22 mg/g of extract; DM leaf: 32.11 ± 3.90 mg/g of extract) was the highest quantified individual compound while trans cinnamic acid was the least quantified individual compound (ethanolic leaf: 7.68 ± 0.55 mg/g of extract; DM leaf: 2.52 ± 0.66 mg/g of extract) in both ethanolic and DM leaf extracts. Similarly, among phenolics tested, eugenol was the highest quantified individual compound (ethanolic leaf: 104.38 ± 1.79 mg/g of extract; DM leaf: 93.11 ± 0.42 mg/g of extract) while gallic acid was the least quantified individual compound (ethanolic leaf: 0.81 ± 0.06 mg/g of extract; DM leaf: 0.76 ± 0.04 mg/g of extract). The detailed results of quantified individual compounds are given in (Table 4) and HPLC chromatograms of ethanolic and DM leaf extracts are given in Figs. 3 and 4.

**Table 4:** Quantity of individual phenolic and non phenolic compounds

Individual compound	(mg/g of extract)	
	Ethanolic leaf	DM leaf
Cinnamyl acetate	44.53 ± 3.22^a^	32.11 ± 3.90^b^
Cinnamaldehyde	8.20 ± 0.25^a^	5.57 ± 0.41^b^
Trans cinnamic acid	7.68 ± 0.55^a^	2.52 ± 0.66^b^
Eugenol	104.38 ± 1.79^a^	93.11 ± 0.42^b^
Catechin	16.48 ± 0.39^a^	18.56 ± 1.10^a^
Epicatechin	7.02 ± 0.43^b^	10.08 ± 0.07^a^
Kaempferol	8.62 ± 1.38^a^	5.62 ± 0.77^a^
Phlorizidin	3.85 ± 0.05^a^	1.20 ± 0.41^b^
4-hydroxy benzoic acid	1.91 ± 0.10^a^	1.27 ± 0.67^a^
Gallic acid	0.81 ± 0.06^a^	0.76 ± 0.04^a^

Results expressed as mean ± SE (*n* = 3 each); Mean values superscripted by different letters in each individual compound were significantly different at *p* < 0.05

*DM* dichloromethane:methanol

## Discussion

Ethanolic and dichloromethane:methanol (DM) extracts of leaf of true cinnamon were studied for anti-inflammatory, growth inhibition and cytotoxicity and antilipidemic potential using well established, widely used, sensitive, specific, reliable and reproducible in vitro bioassays which are frequently used to investigate the health benefits of various medicinal plants including spices [26–30, 32–35]. Further, some selected individual compounds including phenolics and non phenolics were quantified in both ethanolic and DM extracts of leaf of Ceylon cinnamon. Those extracts were used since those extracts were investigated previously for antioxidant and anti-diabetic properties and results highlighted significantly high antioxidant and moderately potent anti-diabetic activities via multiple mechanisms [24, 25].

Investigated both extracts of leaf of Ceylon cinnamon demonstrated anti-inflammatory activity in vitro via nitric oxide (NO) and superoxide (O_2_^•-^) radical scavenging activities and COX1 and COX2 enzyme inhibitory activities. Nitric oxide (NO) and superoxide (O_2_^•-^) are biological radicals and involve in many important physiological functions [36, 37]. However, overproduction of these radicals involves in onset and development of inflammation and inflammation related chronic diseases [38, 39]. Further, the reaction between nitric oxide and superoxide radicals generates cytotoxic and proinflammatory peroxynitrite radical which too have a significant role in inflammation [38]. Therefore, inhibition of nitric oxide and superoxide biological radicals may be certainly important in managing of development and progression of inflammation. The present study reported that investigated both leaf extracts possess nitric oxide and superoxide radical scavenging activities. Further, ethanolic leaf extract showed high activity for both the nitric oxide and superoxide radical scavenging activities (IC_50_ 40.26 ± 0.52 and 696.24 ± 40.02 μg/mL respectively). This is the first study to report scavenging of nitric oxide and superoxide biological radicals by leaf of authenticated Ceylon cinnamon world over. Cyclooxygenases (COXs) are a family of key enzymes in cyclooxygenase pathway and exist in three isozymes such as COX-1, COX-2 and COX-3 [40]. It is reported that induction and over expression of isozyme COX-2 is associated with pathological processes of inflammation [40, 41]. Although nonsteroidal anti-inflammatory drugs (NSAIDs) are effective in inhibiting of COX-2 enzyme reported to have several side effects [42]. Therefore, search of novel cyclooxygenase inhibitors from various natural products are still continuing. However, *Cinnamomum* species as cyclooxygenases inhibitors are poorly investigated to date. Present research reported that Ceylon cinnamon leaf possesses cyclooxygenases inhibition for the first time and highlighted significantly (*p* < 0.05) high COX1 enzyme inhibition compared to COX2 enzyme inhibition. Further, DM leaf extract showed the highest COX1 and COX2 inhibitory activities than ethanolic leaf extract. Like most conventional NSAIDs both extracts inhibit COX1 and COX2 enzymes. Since both extracts have high COX1 inhibitory activity they may have gastrointestinal toxicity effects similar to conventional NSAIDs. However, further investigations are essential to come to a strong conclusion. Previous investigations of *Cinnamomum* species for cyclooxygenases inhibitory activity showed that some phytochemical constituents such as cinnamaldehyde, cinnamic alcohol, cinnamic acid, and eugenol reported to mediate the expression of COX2 gene [43]. Liao et al. [43], reported that cinnamaldehyde had the highest inhibitory effect on expression of COX2 gene among the studied other phytoconstituents such as cinnamic alcohol, cinnamic acid and coumarin. In vivo experiment carried out by Liao et al. [43], further confirmed COX inhibitory activity of cinnamaldehyde is comparable with the potent COX inhibitor, indomethacin. The present research investigated ethanolic and DM leaf extracts had varying quantities of cinnamaldehyde, cinnamic alcohol, cinnamic acid and eugenol. Therefore, observed cyclooxygenases inhibition by leaf of Ceylon cinnamon may be, at least partly, due to these compounds with many other unidentified compounds. Interestingly, the present study reports for the first time that leaf of true cinnamon possesses cyclooxygenases inhibitory activity.

Growth inhibition and cytotoxicity on selected human carcinoma cell lines (MCF 7, HepG2, AN3CA) and inhibition of GST enzyme were the demonstrated anticancer related activity of leaf of Ceylon cinnamon. Further, demonstrated activities were moderate for both the growth inhibition and cytotoxicity and inhibition of glutathione *S*-transferase enzyme. However, ethanolic extract showed high activity than DM extract for all the investigated anticancer related activities. Previous investigations too reported cytotoxicity of Ceylon cinnamon against different carcinoma cell lines as anticancer related activity of Ceylon cinnamon [44]. Further, all previous reports are based on bark of *C. zeylanicum* as the experimental cinnamon sample and to date very few cytotoxic compounds were identified from *Cinnamomum* species [44]. Some of the identified individual compounds from bark extract of *C. cassia* on MCF 7 cells cytotoxicity were trans-cinnamaldehyde and coumarin [45]. Further, eugenol, a major compound in cinnamon leaf oil is too reported to have MCF 7 cells cytotoxicity against human breast cancer cells [46]. In the present study investigated both leaf extracts had varying quantities of trans-cinnamaldehyde and eugenol contents and ethanolic leaf extract had high quantities than DCM:M leaf extract. Therefore, the observed high activity of ethanolic leaf extract for MCF 7 cells cytotoxicity may be at least partly due to these compounds. Similarly, cinnamaldehyde together with cinnamic acid and cinnamyl alcohol reported to possess antiproliferative activity against HepG2 cells [47]. This was reported by Ng and Wu [47], and showed that the order of potency of these three compounds for antiproliferative activity against HepG2 cells were cinnamaldehyde *>* cinnamic acid *>* cinnamyl alcohol. Further, it was reported that the potency of antiproliferative activity of cinnamaldehyde is similar to the anti-cancer drug 5-fluorouracil (IC_50_ cinnamaldehyde and 5-fluorouracil; 9.76 ± 0.67 and 9.57 *±* 0.61 μM respectively). In this study among investigated leaf extracts ethanolic leaf extract of Ceylon cinnamon had high contents of cinnamaldehyde and cinnamic acid. Therefore, observed high cytotoxicity of ethanolic leaf extract on HepG2 cells may be, at least partly, due to the high cinnamaldehyde and cinnamic acid contents. Interestingly this is the first report on growth inhibition and cytotoxicity against human hepatocarcinoma (HepG2) cells of leaf of any *Cinnamomum* species worldwide. Further, to date neither bark nor the leaf of any *Cinnamomum* species reported for cytotoxicity against human endometrial carcinoma. Therefore, this is the first report on growth inhibition and cytotoxicity against human endometrial carcinoma (AN3CA) cells of any *Cinnamomum* species world over. Modulation of glutathione *S*-transferase enzyme is the other anticancer related mechanism demonstrated by both leaf extracts of Ceylon cinnamon. Several previous reports highlighted over expression of GST isozymes in different human carcinomas when compared to corresponding normal tissues. Although, it is an adaptive cellular response to protect vital cellular nucleophiles from drug-induced damage this is thought to be associated with a poor prognosis for response to anticancer treatment of patients [48]. Therefore, to ameliorate these resistances, a rationale has been established to utilize agents that specifically inhibit the various classes of GST as adjuvant in chemotherapy. Present study reports for the first time GST inhibitory activity of leaf of Ceylon cinnamon and ethanolic leaf extract demonstrated significantly (0.05 < p) high GST inhibitory activity than DM leaf extract. Further, this is a novel biological activity exhibited by the Ceylon cinnamon leaf and leaf of any *Cinnamomum* species world over.

Both leaf extracts of Ceylon cinnamon showed antilipidemic activity and activity by inhibition of lipid synthesis, digestion and absorption in vitro. Inhibition of enzyme 3-hydroxy-3-methylglutaryl-coenzyme A (HMG-CoA) reductase is the demonstrated lipid synthesis inhibition by both leaf extracts of Ceylon cinnamon. It is the rate-limiting enzyme in cholesterol and other isoprenoids biosynthesis pathway. Therefore, inhibitors of this enzyme can play a major role in the management of hyperlipidemia [49, 50]. Previous investigations showed that some bioactive phytochemical constituents such as phenolics including proanthocyanidins and some individual compounds such as cinnamate (trans cinnamic acid) and cinnamaldehyde possess HMG-CoA reductase enzyme inhibition [51, 52]. In the present study investigated both leaf extracts had varying quantities of total phenolics [25], proanthocyanidins [24], trans cinnamic acid (ethanol leaf and DCM:M 7.68 ± 0.55 and 2.52 ± 0.66 mg/g of extract respectively) and cinnamaldehyde (ethanol and DCM:M leaf extracts: 8.20 ± 0.25 and 5.57 ± 0.41 mg/g of extract respectively) contents. Therefore, these bioactive phytochemicals may be responsible for inhibition HMG-CoA reductase enzyme in the present study. Further, previous reports on HMG-CoA reductase enzyme inhibitory activity of *Cinnamomum* species are extremely rare except the study reported by Lopes et al. [51], on bark extract of *C. zeylanicum*. Moreover, this is the first report on HMG-CoA reductase inhibitory activity of leaf of any *Cinnamomum* species world over.

Another target area in managing hyperlipidemia is the inhibition of fat digestion and absorption [35, 53]. Pancreatic lipase is the key enzyme responsible for intestinal fat digestion into free fatty acids and glycerol [54]. Therefore, previous reports on antihyperlipidemic activity of natural products were mostly targeted on pancreatic lipase inhibition [35, 53] and anti-obesity agents [54]. This research indicated that both leaf extracts had moderate activity towards lipase enzyme inhibition (leaf extracts: 12.92 ± 0.78 - 19.71 ± 0.97 inhibition at 600 μg/mL). Some of the polyphenolic compounds reported to possess anti-lipase activity included procyanidins, chalcones, flavones, flavonols and tannins [54]. The present research, identified some compounds such as gallic acid (0.76 ± 0.04 - 0.81 ± 0.06 mg/g of extract), kaempferol (5.62 ± 0.77 – 8.62 ± 1.38 mg/g of extract), catechin (16.48 ± 0.39 – 18.56 ± 1.10 mg/g of extract) and epicatechin (7.02 ± 0.43 – 10.08 ± 0.07 mg/g of extract) reported to have lipase inhibition [55]. Our previous publication highlighted that leaf of true cinnamon possess high polyphenolic contents (leaf extracts: 22.91 ± 0.11- 44.57 ± 0.51 mg gallic acid equivalents/g of leaf) [25] including proanthocyanidins (309 ± 3–434 ± 14 mg cyanidin equivalents/g of extract) [24]. Since, polyphenolic compounds such as proanthocyanidins and other antioxidants in leaf may be responsible for the observed lipase inhibition [55, 56]. Further, according to the available literature there are no previous scientific reports on lipase enzyme inhibition by leaf of any *Cinnamomum* species world over. Therefore, this is novel finding may help in managing obesity and hyperlipidemia in the world. Dietary fat absorption in the small intestine is a multi-step process and can be regulated through inhibition of pancreatic cholesterol esterase [57], cholesterol micellization [58, 59] and bile acid binding [35]. Investigated both leaf extracts of true cinnamon had moderate cholesterol esterase and cholesterol micellization inhibitory activities and ethanolic leaf extract showed the highest activity. Cholesterol esterase and cholesterol micellization inhibition by certain polyphenolic compounds is well reported and some of the reported compounds are epicatechin, catechin and gallic acid [56]. Leaf extracts of Ceylon cinnamon also consist of high quantity of polyphenolics [25] and also had varying quantities of catechin, gallic acid, and epicatechin. Therefore, polyphenolic compounds in leaf might have a significant role in absorption of dietary fats in small intestine. Further, to date there are no previous scientific literature on inhibition of cholesterol esterase and cholesterol micellization by leaf of any *Cinnamomum* species world over.

Oxidative stress plays a crucial role in the development of many age-related chronic diseases [60]. Further, oxidative stress and inflammation are closely related and tightly linked pathophysiological processes [17, 61]. Large number of scientific reports highlighted the usefulness of polyphenols in adjuvant therapy for the management of inflammation and chronic diseases [17, 61]. They are primarily the phenolics including catechins, flavonoids, anthocyanins and proanthocyanidins [62–65]. Majority of polyphenols are potential antioxidants [17, 61]. Both ethanolic and DCM:M leaf extracts were rich in polyphenolics and which included phenolics, flavonoids and proanthocyanidins in varying quantities [24, 25]. Further, both leaf extracts demonstrated anti-oxidant activity via multiple mechanisms [25]. Therefore, antioxidants and anti-oxidant activity of polyphenolics in true cinnamon leaf might have some effects for the demonstrated anti-inflammatory, growth inhibition and cytotoxicity and antilipidemic properties.

## Conclusion

In conclusion results showed that leaf of Ceylon cinnamon exhibited a range of medically important bio active properties such as anti-inflammatory, antilipidemic and anticancer related activities. Each bio activity of leaf of Ceylon cinnamon is mediated via multiple mechanisms. The mechanisms included nitric oxide and superoxide radical scavenging activities and COX1 and COX2 enzyme inhibitory activities for anti-inflammatory activity, growth inhibition and cytotoxicity against MCF7, HePG2 and AN3CA human carcinoma cell lines and glutathionase-S-transferase inhibitory activity for anticancer related activity and HMG-CoA reductase, lipase, cholesterol esterase and cholesterol micellization inhibitory activities for antilipidemic activity. Further, leaf extracts of Ceylon cinnamon had varying quantities of bio active phytochemicals where cinnamaldehyde, cinnamic acid, cinnamyl acetate and eugenol may be responsible mostly for the observed bio active properties. Moreover, ethanolic leaf extract showed the highest bio active properties (except COX1 and COX2 inhibitory activities, HMG-CoA reductase and lipase inhibitory activities) and had the highest quantities of quantified bio active phytochemicals (except epicatechin) than DM leaf extract. This is the first study to report a range of medically important bio active properties such as anti-inflammatory, antilipidemic and anticancer related activities of leaf of Ceylon cinnamon and findings help to scientifically validate some of the Sri Lankan traditional medicinal claims of leaf of Ceylon cinnamon. Further, novel findings of the present study will help in developing promising novel anti-inflammatory, anticancer and antilipidemic functional health foods and food supplements, nutraceuticals and cosmeceuticals and application in adjuvant therapy in managing diverse chronic diseases.

## Data Availability

The data used and/or analyzed during this study are available from the corresponding author on request.
